# The impact of obesity on hospitalized patients with COVID-19 infection in the Eastern Province of Saudi Arabia

**DOI:** 10.25122/jml-2022-0033

**Published:** 2022-03

**Authors:** Dania AlKhafaji, Reem Al Argan, Salma Albahrani, Abdulmohsen Al Elq, Waleed Albaker, Mohammed Al-Hariri, Abrar Alwaheed, Safi Alqatari, Alaa Alzaki, Abir Alsaid, Marwan Alwazzeh, Fatimah AlRubaish, Zainab Alelq, Tariq Alsaif, Mohammad Zeeshan, Nada Alzahrani, Abdulrahman Alhusil, Batool Gasmelseed, Fatma Zainuddin, Amani Alhwiesh, Nafie Alrubaish

**Affiliations:** 1. Department of Internal Medicine, College of Medicine, Imam Abdulrahman Bin Faisal University, Khobar, Saudi Arabia; 2. Department of Internal Medicine, King Fahad Military Medical Complex, Dhahran, Saudi Arabia; 3. Department of Physiology, College of Medicine, Imam Abdulrahman Bin Faisal University, Dammam, Saudi Arabia; 4. Department of Medical Education, College of Medicine, Imam Abdulrahman Bin Faisal University, Dammam, Saudi Arabia; 5. Department of Medical Allied Services, Imam Abdulrahman Bin Faisal University, Khobar, Saudi Arabia

**Keywords:** body mass index, outcome, obesity, mortality, severity, COVID-19

## Abstract

This study aimed to assess the association of obesity with the severity and outcome of COVID-19 infection. A retrospective observational study was performed from March to September 2020 in Saudi Arabia. Baseline and laboratory data were collected from the inpatient health record system. The cohort was divided into three groups based on body mass index. Following this, the severity and outcome of COVID-19 disease were analyzed between the three groups. Of the 502 COVID-19 cases included, 244 (48.5%) were obese. Obesity was significantly associated with severe (53.5%) or critical (28%) COVID-19 infection (P<0.001) and a higher need for ICU admission (35.8%, P=0.034). Multivariate analysis showed that overweight/obesity was an independent risk factor of severe (P<0.001) as well as critical COVID-19 infection (P=0.026, respectively) and a predictor of a higher risk of ICU admission (P=0.012). Class I obesity was associated with severe-critical COVID-19 disease (33.6%, P=0.042) compared to other obesity classes. Obesity is an independent risk factor for severe-critical COVID-19 infection and a higher risk of ICU admission. Clinicians should give special attention to such populations and prioritize vaccination programs to improve outcomes.

## INTRODUCTION

In March 2020, the World Health Organization (WHO) announced coronavirus disease (COVID-19) as a global pandemic [1]. As of writing this report, it had affected approximately 269 million cases worldwide, with a total mortality rate of 5 million cases [2]. Saudi Arabia is highly affected by this pandemic. As of December 2021, there are 550,000 affected cases, with a total mortality rate of 8800 cases [3].

The course of COVID-19 infection varies widely among patients [4], and some patients present a higher risk of serious illness and bad outcomes [5]. Those include older patients or patients with co-morbidities like chronic kidney disease (CKD), hypertension (HTN), cardiac diseases, and *diabetes mellitus* (DM) [6].

Several observational studies showed that obese and/or overweight people have more adverse outcomes of COVID-19 disease [7, 8]. For example, Simonnet *et al.* studied the relationship between obesity and COVID-19 infection severity and found that a higher percentage of critical care admissions were among patients with obesity [9]. Similarly, Cai *et al.* reported that patients with obesity had a higher odds ratio (2.42) of developing severe pneumonia [10]. The prevalence of obesity is high in both genders in the Saudi population [11]. In fact, obesity increases over time, with a reported prevalence of 27.6% among the Saudi population [12].

The majority of retrospective studies performed in Saudi Arabia during the same period focused on more complex outcomes such as DM [13, 14], mental health [15], and other risk factors, including vitamin D deficiency [16–18]. These studies were also concentrated in the central region.

Uncovering the association between COVID-19 infection severity and body mass index (BMI) is necessary for treatment. However, published data on this association in Saudi Arabia and the Middle East is scarce. Therefore, the aim of the current study was to assess the impact of obesity on the outcomes and severity of hospitalized patients with COVID-19 infection in Eastern Saudi Arabia.

## MATERIAL AND METHODS

This retrospective observational study was carried out in two tertiary hospitals in the Eastern Province of Saudi Arabia (King Fahd Military Medical Complex and King Fahd Hospital of the University) from March to September 2020. Inclusion criteria included all admitted patients who were ≥18 years, confirmed COVID-19 disease by SARS-COVID-19 PCR test. Exclusion criteria were pregnant women and patients with malignancy and immunodeficiency diseases. First, the cohort was divided according to patients' BMI into three groups: (1) normal weight (BMI 20–24.9 Kg/m^2^), (2) overweight (BMI 25–29.9 Kg/m^2^), and (3) obese (BMI ≥30 Kg/m^2^) [13]. Then, the obese group was further divided into 3 classes: Class I (BMI 30–34.9 Kg/m^2^), Class II (BMI 35–39.9 Kg/m^2^), and Class IIII (BMI ≥40 Kg/m^2^) [19].

### Data collection

Data was collected from the inpatient health record system of the two hospitals and included the following: (1) socio-demographic information (age, gender, and nationality), (2) body weight, height, and calculated BMI, (3) co-morbidities (DM, HTN, CKD, and cardiac diseases), (4) baseline laboratory investigations: D-dimer, erythrocyte sedimentation rate (ESR), ferritin, complete blood count (CBC), C-reactive protein (CRP), lactate dehydrogenase (LDH) and renal profile, (4) COVID-19 severity data classified according to the 2020 Saudi Ministry of Health COVID-19 Management Guidelines [20]. The following criteria were followed in the classification of the severity of COVID-19 disease:

A. Mild-moderate disease: if there is no pneumonia on chest x-ray and no oxygen requirement;
B. Severe disease: reflected by any of the followings: oxygen saturation ≤93% on room air, respiratory rate ≥30/minute, partial pressure of oxygen (PaO_2_)/fraction of inspired oxygen (FiO_2_) <300 or lung infiltrates >50% of the lung field within 24–48 hours and;
C. Critical disease reflected by any of the followings: altered level of consciousness, acute respiratory distress syndrome (ARDS), sepsis, multi-organ failure, or risk factors of cytokine storm syndrome if there are one or more of the followings: Ferritin >600 ug/L at presentation and LDH>250 U/L or high D-Dimer >1 mcg/ml.

The outcomes of COVID-19 infection were determined by the need for intensive care unit (ICU) admission, mechanical ventilation, length of hospital stay, and mortality. Then, the outcomes and severity of COVID-19 disease were compared and analyzed between the three study groups. After that, we compared the outcomes and severity of COVID-19 between the three classes of obesity.

### Statistical Analysis

Data were analyzed using the IBM SPSS version 24 software. The distribution of the categorical variables was summarized using percentages, and continuous variables were presented using median and interquartile range (IQR). Associations between categorical variables were assessed using Fisher exact test or Chi-square test. Multiple regression was used to assess the predictors of COVID-19 infection outcomes and severity. For all the analyses, the significance level was set at 5% (≤0.05).

## RESULTS

### Demographic characteristics and co-morbidities

In total, 502 confirmed COVID-19 cases were included in the study. There were 176 (35.1%) females and 326 (64.9%) males. The majority, 369 (73.7%), were Saudis. The majority of patients, 217 (43.2%), were aged between 41–60 years, followed by 151 patients (30.1%) between 61–80 years, 111 (22.1%) cases between 20–40 years, 14 (2.8%) cases were above >80 years and 9 (1.8 % )cases were < 20 years.

Out of 502 cases, 244 (48.6%) were obese, 149 (29.7%) were overweight, and 109 (21.8%) had normal weight. A total of 202 (40.2%) had severe COVID-19 disease, 148 (29.5%) mild-moderate disease, and 152 (30.3%) were critical cases. The most prevalent co-morbidities were DM (39.9%) followed by hypertension (38.9%), CKD (24.35%), and cardiac disease (18.76%) (Table 1).

**Table 1.** Demographic data of participants.

		Frequency	Percentage
**Gender**	**Male**	326	64.9
	**Female**	176	35.1
**Nationality**	**Saudi**	369	73.5
	**Non-Saudi**	133	26.5
**Age (years)**	**<20**	9	1.8
	**20–40**	111	22.1
	**41–60**	217	43.2
	**61–80**	151	30.1
	**>80**	14	2.8
**BMI**	**Normal weight**	109	21.8
	**Overweight**	149	29.7
	**Obese**	244	48.6
**Classes of obesity**	**Class I**	135	55.3
	**Class II**	58	23.8
	**Class III**	51	20.9
**Severity**	**Mild-Moderate**	148	29.5
	**Severe**	202	40.2
	**Critical**	152	30.3
**Comorbidities**	**DM**	200	39.92
	**HTN**	195	38.92
	**Cardiac disease**	94	18.76
	**CKD**	122	24.35

DM – *diabetes mellitus*; HTN – hypertension; BMI – body mass index; CKD – chronic kidney disease.

### Comparison of laboratory findings between the study groups

The study results displayed that blood urea nitrogen (BUN), lymphocyte, and ferritin were significantly higher in patients with obesity (P<0.05). In addition, CRP, LDH, D-dimer, ESR, and neutrophils were higher in patients with obesity, but they did not reach statistical significance. All other parameters were statistically similar in the three groups (Table 2).

**Table 2.** Laboratory findings among COVID-19 groups according to BMI.

Laboratory parameter	Normal range	Median (IQR)			P-values
		Normal weight	Overweight	Obese	
**WBCs**	(4.0–10.0 k/ul)	7.3 (3.8–11.6)	8.9 (4.4–14.9)	9.8 (5.4–14.7)	0.052
**Hgb**	Females (12.0–16.0 g/dl)	12.8 (11.9–14)	13 (12.7–14.7)	13.7 (11.9–14.4)	0.244
	Males (13.0–18.0 g/dl)				
**Platelets**	(140–450)	196.5 (149.5–252)	190 (158–247)	212 (155–260)	0.858
**Neutrophil**	(2.0–7.5 k/uL)	4.6 (2.4–8.3)	7 (3–10.9)	7.1 (3.4–11.8)	0.053
**Lymphocyte**	(1.0–5.0 k/ul)	1.1 (0.8–2)	1 (0.7–1.3)	1.3 (0.9–1.7)	**<0.001**
**BUN**	(7–26 mg/dl)	12.6 (9–18.9)	13.7 (10–19)	15 (11–23)	**0.003**
**Creatinine**	(0.6–1.2 mg/dl)	0.9 (0.7–1.2)	1 (0.8–1.2)	1 (0.8–1.3)	0.066
**Na**	(136–146 mEq/L)	135 (131–137.5)	135 (133–139)	136 (132–137)	0.271
**K**	(3.5–5.1 mEq/L)	4 (3.8–4.5)	4.2 (3.6–4.5)	4.2 (3.8–4.5)	0.062
**CO_2_**	(20–31 mEq/L)	23 (21–26)	23 (21–26)	24 (20–26)	0.766
**LDH**	(81–234 U/L	376 (297.5–515)	376 (281–608)	411 (304–570)	0.557
**ESR**	(0–20 mm/hour)	48.5 (26.5–75)	52 (39–72)	52 (32–67)	0.811
**CRP**	(0.1-0.5 mg/dl)	8.3 (3.6–17.7)	8.9 (4.1–18.8)	10.2 (5.2–18.4)	0.535
**D-Dimer**	≤0.5 ug/mL	1 (0.5–3.4)	0.8 (0.5–1.4)	1.1 (0.6–1.7)	0.986
**Ferritin**	(21.81–274.66 ng/ml)	325 (131–741)	548 (214–1311.1)	591 (270–1154)	**0.003**

WBCs – white blood cells; Hgb – Hemoglobin; Na – Sodium; K – Potassium; Cl – chloride; CO_2_ – Carbon dioxide; LDH – Lactate dehydrogenase; ESR – Erythrocyte sedimentation rate; CRP – C-reactive protein; bold – Significant value.

### Association between co-morbidities and the outcomes and severity of COVID-19 infection

Univariate analysis indicated a significant association between cardiac disease and critical COVID-19 infection (P=0.021). However, there was no association between the severity of COVID-19 pneumonia with gender, age, and other co-morbidities (Table 3).

**Table 3.** Association between severity of COVID-19 infection with co-morbidities, age, and gender.

Variable		Severity			P-value
		Mild-Moderate	Severe	Critical	
**Gender**	**Male**	93 (28.5%)	129 (39.6%)	104 (31.9%)	0.53
	**Female**	55 (30.7%)	73 (40.8%)	48 (26.8%)	
**Age**	**<20**	3 (33.3%)	5 (55.6%)	1 (11.1%)	0.21
	**20–40**	36 (32.4%)	51 (45.9%)	24 (21.6%)	
	**41–60**	57 (26.3%)	90 (41.5%)	70 (32.3%)	
	**61–80**	46 (30.5%)	50 (33.1%)	55 (36.4%)	
	**>80**	6 (42.9%)	6 (42.9%)	2 (14.3%)	
**Co-morbidities**	*Diabetes mellitus*	54 (27%)	92 (46%)	54 (27%)	0.3
	**Hypertension**	51 (26.2%)	82 (42.1%)	62 (31.8%)	0.5
	**Cardiac disease**	27 (28.7%)	28 (29.8%)	39 (41.5%)	**0.021**
	**Chronic kidney disease**	31 (25.4%)	47 (38.5%)	44 (36.1%)	0.3

Univariate analysis test. Bold – Significant value.

### Association between obesity and the outcomes and severity of COVID-19 infection

**Figure 1.**
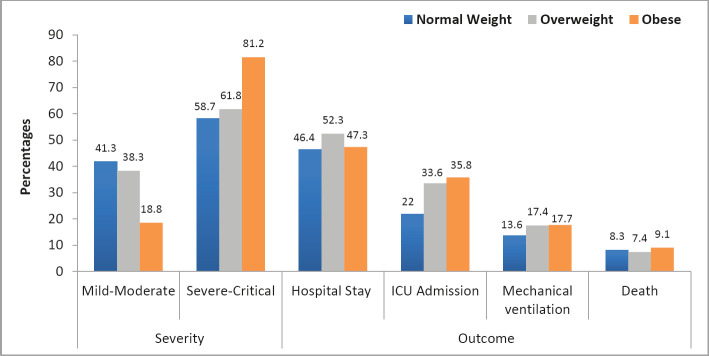
Association between outcomes and severity of COVID-19 infection with the three study groups. Univariate analysis test. ICU – Intensive care unit.

Univariate analysis (Table 4, Figure 1) revealed that obesity was significantly associated with critical (27.9%) or severe (53.3%) COVID-19 infection (P<0.001). Examining the association of body weight based on BMI with different severity classes of COVID-19 infection revealed that mild-moderate disease was associated with normal weight (51.4%; P<0.0001).

**Table 4.** Association between outcomes and severity of COVID-19 infection according to BMI.

	**Variable**	**The study groups based on BMI**			**P-Values**
		**Normal weight**	**Overweight**	**Obese**	
**Severity**	**Mild-moderate**	45 (41.3%)	57 (38.3%)	46 (18.8%)	**0.001**
	**Severe**	29 (26.6%)	43 (28.9%)	130 (53.3%)	
	**Critical**	35 (32.1%)	49 (32.9%)	68 (27.9%)	
**Severity criteria**	**Mild-Moderate**				
	**No oxygen requirement and no pneumonia on chest x-ray**	56 (51.4%)	64 (42.9%)	28 (11.5%)	**<0.0001**
	**Severe**				
	**Respiratory rate>30/minute**	13 (11.9%)	20 (13.4%)	133 (54.7%)	**<0.0001**
	**Oxygen saturation <93% on room air**	17 (15.6%)	35 (23.5%)	119 (49%)	**<0.0001**
	**PaO_2_/FiO_2_<300**	7 (6.4%)	20 (13.4%)	57 (23.5%)	**<0.0001**
	**Lung infiltrates >50% of lung field within 24–48 hours**	8 (7.3%)	13 (8.7%)	127 (52.3%)	**<0.0001**
	**Critical**				
	**Acute respiratory distress syndrome**	16 (14.7%)	24 (16.1%)	45 (18.5%)	0.64
	**Sepsis**	11 (10.1%)	16 (10.7%)	37 (15.2%)	0.277
	**Altered level of consciousness**	11 (10.1%)	16 (10.7%)	21 (8.6%)	0.778
	**Multi organ failure**	5 (4.6%)	8 (5.4%)	18 (7.4%)	0.528
	**Cytokine Storm: Ferritin>600 ug/L at presentation & LDH>250 U/L**	12 (16.5%)	23 (15.4%)	15 (6.2%)	**0.003**
	**Cytokine Storm: D-Dimer >1 mcg/ml**	17 (15.6%)	12 (8.1%)	23 (9.5%)	0.118
**Outcome**	**Hospital stay** **Median (IQR) (days)**	7 (3–11)	8 (4–14)	7 (4–12)	0.35
	**ICU Admission n (%)**	24 (22)	50 (33.6)	87 (35.8%)	**0.034**
	**Mechanical ventilation n (%)**	15 (13.8%)	26 (17.4%)	43 (17.7%)	0.7
	**Death n (%)**	9 (8.3%)	11 (7.4%)	22 (9.1%)	0.8

Univariate analysis test. BMI – Body mass index; PaO_2_ – Partial pressure of oxygen; FiO_2_ – Fraction of inspired oxygen; LDH – lactate dehydrogenase; IQR – Interquartile range; ICU – Intensive care unit; N – number; % – percentage; bold – Significant value.

However, obesity was associated with all severe class criteria (P<0.0001) and cytokine storm syndrome with high LDH and ferritin from the critical class criteria (P=0.003) (Table 4, Figure 1). However, other outcome variables were not significant among the three groups (Table 4, Figure 1). In multivariate analysis (Table 5), higher BMI levels (including overweight and obese groups) were an independent risk factor for both severe (OR 2.4; P<0.001) and critical (OR 1.4; P=0.026) COVID-19 disease. Looking at the association between outcome and BMI, our results revealed that a higher BMI was associated with a greater need for ICU admission (35.8%, P=0.034). Furthermore, multivariate analysis showed that higher BMI was an independent risk factor for ICU admission (OR 1.0; P=0.012) (Table 5).

**Table 5.** Association between outcomes and severity of COVID-19 infection with BMI, co-morbidities, age, and gender.

Variable	Severity				Outcome							
	Severe		Critical		*Hospital stay		ICU admission		Mechanical ventilation		Death	
	OR (95% CI)	P-value	OR (95% CI)	P-values	OR (95% CI)	P-values	OR (95% CI)	P-values	OR (95% CI)	P-values	OR (95% CI)	P-value
**Age**	0.8 (0.5–1.1)	0.12	1.03 (0.7–1.5)	0.8	1.72 (1.3–2.27)	**<0.001**	2.07 (0.4–10.7)	**<0.0001**	1.63 (0.8–2.9)	**0.012**	2.15 (0.5–11)	**0.003**
**Gender**	1.1 (0.7–1.6)	0.8	1.3 (0.8–2.1)	0.2	2.11 (1.37–3.25)	**0.001**	2.12 (0.5–11.7)	**0.003**	1.530 (0.9–2.6)	0.159	1.236 (0.7–2.2)	0.576
**DM**	1.4 (0.8–2.3)	0.22	0.6 (0.3–1.1)	0.9	1.31 (0.84–2.04)	0.235	1.184 (0.5–2.6)	0.482	1.239 (0.7–2.2)	0.476	1.254 (0.7–2.2)	0.551
**HTN**	1.3 (0.7–2.2)	0.4	1.01 (0.6–1.7)	0.6	0.85 (0.53–1.37)	0.514	0.59 (0.4–1.6)	**0.035**	0.48 (0–3.1)	**0.018**	0.32 (0.1–4.1)	**0.007**
**Cardiac disease**	0.6 (0.3–1.3)	0.2	1.2 (0.6–2.3)	0.5	0.63 (0.35–1.12)	0.118	1.287 (0.7–2.2)	0.410	0.540 (0.4–1.6)	0.062	0.620 (0.5–2.6)	0.233
**CKD**	1.3 (0.7–2.3)	0.4	1.9 (1.1–3.5)	**0.028**	2 (1.25–3.2)	**0.004**	1.079 (0.4–2.8)	0.764	0.737 (0.4–2.6)	0.304	1.338 (0.8–3.2)	0.468
*BMI	2.4 (1.8–3.2)	**<0.001**	1.4 (1–2)	**0.026**	1.01 (0.98–1.04)	0.582	1 (0.2–2.4)	**0.012**	1.009 (0.2–1.4)	0.647	1.010 (0.1–1.9)	0.709

Multivariate analysis test. OR–Odds ratio; CI–Confidence Interval; DM–Diabetes mellitus; HTN–hypertension; BMI–Body mass index; CKD–chronic kidney disease. *Hospital stay: we used a cutoff of 7 days correlated with the mean hospital stay of COVID-19 disease patients in previous studies [43]. Significant values are shown in bold.

In addition, multivariate analysis (Table 5) showed that aging was significantly associated with ICU admission (OR 2.07; P<0.0001), longer hospitalization (OR 1.72; P<0.001), mechanical ventilation (OR 1.63; P=0.012), and death (OR 2.15; P=0.003) while male gender was associated with longer hospitalization (OR 2.11; P<0.001) and a higher need for ICU admission (OR 2.12; P=0.003). Furthermore, CKD was significantly associated with critical COVID-19 disease (OR 1.9; P=0.028) and longer hospital stay (OR 2.0; P 0.004). In addition, HTN was significantly associated with a higher requirement for ICU admission (OR 0.59; P 0.035), mechanical ventilation (OR 0.48; P=0.018) and death (OR 0.32; P=0.007).

### Comparison of age, gender, and different classes of obesity on the outcomes and severity of COVID-19 infection

Older age >40 years was associated with a higher risk of ICU admission, death, and longer hospitalization (P<0.05). We also found that male gender was associated with longer hospitalization time (P=0.02). In addition, obesity (class I) was associated with a higher risk of severe-critical COVID-19 infection (33.3%, P=0.042). However, there was no difference between obesity classes in the outcome variables, including the need for mechanical ventilation, ICU admission, length of hospitalization, or death (Table 6).

**Table 6.** The effect of gender, age, and obesity classes on the outcomes and severity of COVID-19 infection.

Variable	Severe-Critical	*Hospital Stay	ICU Admission	Mechanical ventilation	Death
**Age**					
≤40 years (n=56)	48 (85.7%)	19 (35.2%)	12 (21.4%)	5 (8.9%)	1 (1.8%)
>40 years (n=188)	150 (79.8%)	96 (51.1%)	75 (39.9%)	38 (20.2%)	21 (11.2%)
P-Value	0.35	**0.03**	**0.01**	0.050	**0.03**
**Gender**					
Male (n=146)	118 (80.8%)	77 (52.7%)	56 (38.4%)	27 (18.5%)	13 (8.9%)
Female (n=98)	80 (81.6%)	38 (39.2%)	31 (31.6%)	16 (16.3%)	9 (9.2%)
P-Value	0.96	**0.02**	0.265	0.63	0.713
**Obesity class**					
Class1 "BMI: 30–34.9 kg/m^2^"	45 (33.3%)	62 (45.9%)	49 (35.6%)	25 (18.5%)	12 (8.9%)
Class 2 "BMI: 35–39.9 kg/m^2^"	11 (19%)	29 (50%)	21 (36.2%)	13 (22.4%)	8 (13.8%)
Class 3 "≥40 kg/m^2^"	12 (23.5%)	24 (47.1%)	17 (33.3%)	5 (9.8%)	2 (3.9%)
P-Value	**0.042**	0.865	0.9	0.207	0.202

*Hospital stay: we used a cutoff of 7 days correlated with the mean hospital stay of COVID-19 disease patients in previous studies [43]. Bold – Significant value.

## DISCUSSION

Our study shows the following key results. First, out of 502 COVID-19 patients, a significant proportion was either obese (48.6%) or overweight (29.7%), constituting at least 70% of our cohort, which indicates a high risk of hospitalization among overweight/obese patients. Second, higher BMI (including being overweight and obese) was associated with severe-critical COVID-19 in univariate analysis, and the multivariate analysis confirmed it as an independent risk factor for severe-critical COVID-19 disease. Third, obesity and/or being overweight are independent risk factors for ICU admission. Next, obesity (class I) was associated with a higher risk of severe-critical COVID-19 infection in comparison with other obesity classes. However, this result is difficult to interpret in our study since most of our obese group had class I obesity. In addition, male gender and older age group >40 years in the obese group were associated with worse outcomes. Moreover, obesity was associated with increased levels of lymphocytes, BUN, and ferritin. Finally, we found that older age, male gender, CKD, and HTN were associated with a bad prognosis of COVID-19 disease.

Our study is in line with previous reports showing a significant association between higher BMI and hospitalization time, more severe COVID-19 disease, and worse outcomes, primarily the higher need for ICU admission, as we found in our report [21–27]. For example, Melebari *et al.* looked at 369 COVID-19 cases from Saudi Arabia and found that 45.8% were obese, indicating a greater risk of hospitalization among obese patients similar to our findings [28]. In addition, patients with obesity had a higher risk of pneumonia and ARDS [28]. Gao *et al.* found that higher BMI is associated with a 3-fold increased risk of severe COVID-19 pneumonia in addition to a 12% increase in the risk of severe COVID-19 infection with each 1 unit increase in BMI [29]. Furthermore, a meta-analysis of 46 studies conducted by Cai *et al.* involving 625,000 COVID-19 patients found that patients with obesity had a significantly increased risk of infection (OR 2.73), clinically severe disease (OR 3.81), hospitalization (OR 1.72), mechanical ventilation (OR 1.66), ICU admission (OR 2.25) and mortality (OR 1.61) [30]. Similarly, Rottoli *et al.* reported a higher risk of ICU admission and respiratory failure at BMI 30-34.9 kg/m^2^ with an increased risk of death at BMI ≥35 kg/m^2^ [27]. Moreover, a large retrospective cohort review analysis that included 770 COVID-19 infected patients from New York concluded that around 35% of obese patients appeared to have a greater risk of ICU admission than normal-weight patients [26]. Simonnet *et al.* reported that almost half (47.5%) of the COVID-19 patients who were admitted to ICU had BMIs of ≥30 kg/m^2^ [9]. Another study found a linear increase in the risk of severe COVID-19 disease at a BMI ≥23 kg/m^2^, leading to hospital admission, ICU admission, and death [31].

Multiple mechanisms could explain the severe COVID-19 disease in obese individuals. First, obesity has been linked to disruptions in lymphoid tissue integrity and leukocyte development [32], leading to a disturbed immune system. This would make those patients less responsive to vaccinations, antivirals, and antimicrobials [33]. Second, obese patients have chronically low adiponectin and high leptin levels, which can also lead to a faulty immunological response [34]. Third, obesity decreases respiratory muscle compliance, increases airway resistance, impairs gas exchange, and reduces lung volume [35], which could alter their respiratory profiles during the infection and may even put them at risk of several respiratory complications [36]. Fourth, obesity is commonly linked with obstructive sleep apnea [37], leading to airway obstruction, increased abdominal pressure, and limited chest expansion, making those patients at a higher need for mechanical ventilation [38]. Fifth, the negative impact of obesity (vitamin K deficiency [39], endothelial dysfunction [40], upregulation of plasminogen activator inhibitor 1 [41], and prothrombotic state) [42] on blood clotting mechanisms may contribute to the bleeding as well as the progression of thrombus formation. Finally, angiotensin-converting enzyme II, a binding receptor of the COVID-19 [43], is highly expressed in adipose tissue and could serve as a virus reservoir [44].

Our findings show that obesity was associated with increased levels of lymphocytes, ferritin, and BUN. High circulating ferritin and BUN concentrations were shown in previous evidence to be predictors of the outcomes and severity of COVID-19 infection [45]. This could be one of the factors explaining the worse severity and outcome seen in such a population. Similarly, a retrospective study showed that maximal CRP and ferritin levels during hospitalization were higher in obese than normal-weight COVID-19 patients [46]. Other markers of severity, including D-dimer, LDH, CRP, and ESR, were higher in obese patients in our study but were statistically insignificant.

Similar to previously reported results, our findings indicated that male gender, older age, CKD, and HTN were associated with a bad prognosis of COVID-19 disease [6, 47, 48].

The strengths of our study are the representative sample size and the fact that the sample was taken from double tertiary care centers in the region. In addition, it helps to better understand the behavior of the emerging COVID-19 outbreak in patients with obesity who constitute a large percentage of the population in the region. One of the limitations of this study is that it was concentrated in the Eastern province, Saudi Arabia, which limits the generalizability of our findings. Second, the present study was retrospective which could result in bias.

## CONCLUSION

A greater percentage of hospitalized COVID-19 patients are either overweight or obese. In addition, obesity is an independent risk factor for severe-critical COVID-19 infection and a predictor of the need for ICU admission. As a result, clinicians should give special attention to such populations and prioritize vaccination programs to improve their outcomes.

## ACKNOWLEDGMENTS

### Conflict of interest

The authors declare no conflict of interest.

### Ethical approval

This study was approved by the local Ethics Board in the two hospitals (King Fahd Military Medical Complex, IRB-2020-01-246 and King Fahd Hospital of the University, (AFHER-IRB-2021-010).

### Consent to participate

Written informed consent was taken from all patients.

### Authorship

MA is the corresponding author and was in charge of the manuscript concept and manuscript revision and submission. DA, RA, SA, AALQ, FA, and WA contributed to the methodology and wrote the original draft. AALW, SALQ, AALZ, AALS, MALW, ZA, TA, MZ, NA, and AALH contributed to data collection and curation. BG, FZ, AALHW, and NA contributed to data analysis.
